# Efficacy and Safety of Prolonged Magnesium Sulfate Infusions in Children With Refractory Status Asthmaticus

**DOI:** 10.3389/fped.2022.860921

**Published:** 2022-06-09

**Authors:** Khalid W. Taher, Peter N. Johnson, Jamie L. Miller, Stephen B. Neely, Neha Gupta

**Affiliations:** ^1^Department of Pharmacy: Clinical and Administrative Sciences, University of Oklahoma College of Pharmacy, Oklahoma City, OK, United States; ^2^Office of Instruction, Assessment, and Faculty/Staff Development, University of Oklahoma College of Pharmacy, Oklahoma City, OK, United States; ^3^Department of Pediatrics, Division of Critical Care Medicine, University of Oklahoma College of Medicine, Oklahoma City, OK, United States

**Keywords:** magnesium, continuous infusion, status asthmaticus, children, pediatric intensive care unit

## Abstract

**Objectives:**

There is a paucity of data on the use of intravenous magnesium sulfate infusion in children with refractory status asthmaticus. The purpose of this study was to evaluate the efficacy and safety of prolonged magnesium sulfate infusion as an advanced therapy.

**Methods:**

This is a single center retrospective study of children admitted to our pediatric intensive care unit (PICU) with status asthmaticus requiring continuous albuterol. Treatment group included patients receiving magnesium for ≥4 h and control group included those on other therapies only. Patients were matched 1:4 based on age, sex, obesity, pediatric index of mortality III and pediatric risk of mortality III scores. Primary outcomes included PICU length of stay (LOS) and mechanical ventilation (MV) requirement. Secondary outcomes included mortality, extracorporeal membrane oxygenation (ECMO) requirement, analyses of factors associated with PICU LOS and MV requirement and safety of magnesium infusion. Logistic and linear regressions were employed to determine factors associated with MV requirement and PICU LOS, respectively.

**Results:**

Treatment and control groups included 27 and 108 patients, respectively. Median initial infusion rate was 15 mg/kg/hour, with median duration of 28 h. There was no difference in the MV requirement between the treatment and control groups [7 (25.9%) vs. 20 patients (18.5%), *p* = 0.39]. Median PICU LOS and ECMO use were significantly higher in treatment vs. control group [(3.63 vs. 1.09 days, *p* < 0.01) and (11.1 vs. 0%, *p* < 0.01), respectively]. No mortality difference was noted. On regression analysis, patients receiving ketamine and higher prednisone equivalent dosing had higher odds of MV requirement [OR 19.29 (95% CI 5.40–68.88), *p* < 0.01 and 1.099 (95% CI 1.03–1.17), *p* < 0.01, respectively]. Each mg/kg increase in prednisone equivalent dosing corresponded to an increase in PICU LOS by 0.13 days (95% CI 0.096–0.160, *p* < 0.01). Magnesium infusions were not associated with lower MV requirement or lower PICU LOS after controlling for covariates. Fourteen (51.9%) patients in the treatment group had an adverse event, hypotension being the most common.

**Conclusion:**

Magnesium sulfate infusions were not associated with MV requirement, PICU LOS or mortality.

## Introduction

In the United States of America, children with asthma experience >70,000 hospitalizations and ~700,000 emergency department (ED) visits for asthma exacerbation or status asthmaticus (1). Children with status asthmaticus have high morbidity and healthcare costs and are frequently admitted to the pediatric intensive care unit (PICU) (2). To achieve a rapid improvement in severe asthma exacerbations, concomitant administration of short acting beta-2 agonist and steroids with or without ipratropium is recommended. The National Asthma Education and Prevention Program Coordinating Committee and Global Initiative for Asthma guidelines also recommend the use of a single-dose intravenous (IV) magnesium sulfate of 25–75 mg/kg (maximum of 2 grams/dose) over 20 min in children with asthma exacerbation in the ED with refractory clinical manifestations 1 h after receipt of oral/IV corticosteroids and repeated doses of beta-2 agonists (2, 3). This single dose of magnesium sulfate has been associated with improvement of pulmonary function and decreased odds of hospital admission when administered to children in the ED (2, 4). Other recommended therapies for refractory status asthmaticus include ketamine, terbutaline, and/or aminophylline infusions and heliox. Use of a prolonged IV magnesium infusion for management of refractory asthma exacerbation has also been described in the literature (5).

Eight reports including 447 children have evaluated the use of a prolonged magnesium infusion over ≥1 h (6–13). Most of these studies (*n* = 261; 58.4%) focused on the use of magnesium infusions in the PICU, included patients receiving magnesium sulfate for >4 h (299; 66.9%), and did not evaluate both efficacy and safety. As a result, there remains an existing gap in the literature on the safety and efficacy of prolonged magnesium sulfate infusions. The purpose of this study was to evaluate the efficacy and safety of these prolonged magnesium infusions as an advanced therapy when compared to children receiving other therapies only (terbutaline, aminophylline, ketamine and/or heliox) for refractory status asthmaticus.

## Materials and Methods

### Study Design and Population

This is a retrospective cohort study of children admitted to our 34-bed PICU at a tertiary care academic center from January 1, 2013 to August 31, 2020 with diagnosis of status asthmaticus. Patients were identified using ICD-9 or ICD-10 codes for diagnosis of “asthma” in the Virtual Pediatric Systems (14), and the hospital electronic medical record, Meditech^®^ (Medical Information Technology, Inc., Westwood, MA). Patients were included in the study if they were >37 weeks postmenstrual age and <18 years of age at the time of admission and received continuous albuterol. Patients were included in the treatment group if they received a magnesium sulfate infusion ≥4 h for refractory status asthmaticus. The control group consisted of patients that did not receive magnesium sulfate infusions; controls were matched in a 1:4 fashion using propensity scoring based on age, biological sex, obesity status, pediatric index of mortality (PIM) III and pediatric risk of mortality (PRISM) III scores. Obesity has been previously associated with a significantly higher rate of hospital admissions and a longer hospital and PICU length of stay (LOS) compared with normal weight children (15, 16). Patients were excluded if they received magnesium infusions for indications other than status asthmaticus or if they had incomplete medical records. The study was approved by our institutional review board and waiver of consent was obtained.

### Data Collection and Study Objectives

Demographics and clinical data, including vital signs, age, weight, sex, PIM III and PRISM III scores, PICU LOS, non-invasive and invasive mechanical ventilation (MV) use and use of extracorporeal membrane oxygenation (ECMO), were collected for the treatment and control groups. Non-invasive ventilation included high flow nasal canula, continuous positive airway pressure, and/or bilevel positive airway pressure. Obesity status was determined based on the Centers for Disease Control and prevention calculator for children ≥2 years of age that calculated body mass index (BMI) for age and sex (17). The World Health Organization criterion was used for defining obesity in children <2 years of age (18). Children ≥2 years of age were classified as obese if BMI was ≥95th percentile, while children <2 years of age were classified as obese if weight-for-length was ≥97.7th percentile.

All asthma therapies received by the patients were collected. Corticosteroid total dose was converted and reported as total prednisone dosing equivalents in milligrams (mg) (19). It was also noted if the treatment and control groups received additional advanced treatment options for refractory status asthmaticus. At the time of this study, the advanced treatment options in our PICU's status asthmaticus protocol included terbutaline, aminophylline, ketamine, heliox and magnesium sulfate infusions. Our protocol includes guidance for dosing and monitoring of these therapies. However, selection of agents was based on clinician discretion, and patients may have been initiated on ≥1 therapy based on their clinical status. For magnesium sulfate infusions, the protocol is listed in Supplementary Appendix I. Per the protocol, serum magnesium concentrations were assessed every 4 h, and the magnesium sulfate infusions were titrated to a target goal of 4–6 mg/dL. For the treatment group, the number of patients with hypermagnesemia (serum magnesium level >6 mg/dL), and adverse events (i.e., hypotension, flushing, nausea/vomiting, and infusion related reactions) were collected. Hypotension was defined as systolic blood pressure (SBP) <60 mm Hg in term neonates, SBP <70 mm Hg in infants from 1 to 12 months, SBP <70 mm Hg + (2 × age in years) in children >1 to 10 years, and SBP <90 mm Hg in children over 10 years old (20). Magnesium sulfate data included infusion doses (mg/kg/hour), duration of magnesium infusion (hours), additional magnesium boluses administered (mg and mg/kg), and serum magnesium concentrations (mg/dL).

### Outcomes

Primary outcomes included comparisons of PICU LOS and MV requirement between the treatment and control groups. Secondary outcomes included comparisons of the duration of MV and non-invasive ventilation, patients requiring ECMO and PICU mortality between the treatment and control groups. Another secondary outcome was to determine factors associated with PICU LOS and MV requirement. Additional secondary outcomes focused on a description of the magnesium sulfate infusion dosing regimen and its safety. Last, a sub-analysis was performed for the treatment group to compare the outcomes among children who received magnesium infusions as their first advanced therapy vs. those who received magnesium infusions after another advanced therapy (i.e., 2nd, 3rd, or 4th line).

### Statistical Analysis

Descriptive and inferential statistics were employed. Categorical variables were compared using Chi-square test and were reported as frequency (percentage). Continuous data were compared using independent *t*-test or Mann-Whitney *U*-test depending on if data was normally distributed or not and were reported as mean (standard deviation) or median (interquartile range). Shapiro-Wilk tests were used to determine distributional assumptions. Propensity score matching was conducted using the Matchlt package version 4.3.2 in the R statistical program version 4.1.2 (R Foundation for Statistical Computing, Vienna, Austria). Logistic and linear regressions were employed to determine the associations for MV requirement and PICU LOS, respectively, while controlling for independent variables including continuous albuterol duration, cumulative prednisone equivalent dosing, ketamine exposure and magnesium infusion exposure. A generalized estimating equation was added to the regression models to account for matching *via* propensity scores. Model estimates and 95% confidence intervals are reported for models. Data management and analyses were conducted using SAS software version 9.4 (Statistical Analysis System, Cary, NC) with the alpha set at 0.05.

## Results

A total of 921 patients with asthma were identified during the study period. Sixty of these patients were initiated on magnesium sulfate; however, thirty-three were excluded as they received magnesium sulfate for <4 h. Twenty-seven patients were included in the treatment group. A total of 861 patients were eligible to be considered as controls. There were 516 of these patients who did not receive continuous albuterol and were excluded, leaving 345 remaining eligible patients. After 1:4 ratio propensity score matching was performed, 108 patients were included in the control group.

Baseline demographics of the 135 patients (27 in the treatment group and 108 in the control group) are presented in Table 1. The median age at PICU admission for the treatment and control groups were 8.5 and 9.05 years, respectively, *p* = 0.53. There was no difference in the number of patients classified as obese between the groups, *p* = 0.50. There was no statistical difference in the median PIM III and PRISM III scores or race/ethnicity between the treatment and control groups.

**Table 1:** Patient demographics at PICU admission comparing treatment and control groups (*n* = 135).

**Variables**	**Treatment group (*n* = 27)**	**Control group (*n* = 108)**	***p*-value**
	**Number (%) or Median (IQR)**		
Age (years)	8.5 (6.23–11.94)	9.05 (6.24–13.56)	0.53^a^
Weight (kg)	32.5 (24–55)	39.8 (20.6–63.8)	0.55^a^
**BMI^b^**			
BMI (kg/m^2^)	19.8 (16.8–22.6)	19 (15.9–25.4)	0.92^a^
BMI percentile for age and sex	83.9 (58.6–96.1)	80.0 (44.8–95.7)	0.57^a^
Weight-for-length percentile	99.9^c^	80.0 (44.8–95.7)	
Obesity status	9 (33.3)	29 (26.9)	0.50^d^
Male gender	16 (59.3)	59 (54.6)	0.67^d^
Race/ethnicity			0.14^d^
African American	15 (55.6)	48 (44.4)	
White/Caucasian	6 (22.2)	24 (22.2)	
American Indian/Alaska Native	2 (7.4)	6 (5.6)	
Hispanic	2 (7.4)	4 (3.7)	
Asian American	0 (0)	2 (1.9)	
Mixed	2 (7.4)	2 (1.9)	
Unknown/unspecified	0 (0)	22 (20.4)	
**Mortality scores**			
PIM III	−4.66 (−6.12 to −4.53)	−5.29 (−6.05 to −4.60)	0.98^a^
PRISM III	5 (0–10)	4 (0–7)	0.55^a^

*IQR, Interquartile range; BMI, Body mass index; PIM III, Pediatric Index of Mortality III; PRISM III, Pediatric Risk of Mortality III*.

a *Wilcoxon two-sample test*.

b *BMI assessed in 26 children ≥2 years of age*.

c *Weight-for-length assessed in one child <2 years of age*.

d *Chi-square test*.

All patients in the treatment group and 88 (81.5%) patients in the control group received ≥1 magnesium sulfate bolus dose in either the ED and/or PICU (Table 2). There was statistical difference in the number of patients receiving magnesium sulfate boluses, number of boluses received, and median cumulative dose (mg and mg/kg) between the treatment and control groups. In addition, general characteristics of the magnesium sulfate infusion regimen and concentrations are reported in Table 2. The overall median rate to achieve the target serum concentration was 19.9 mg/kg/h, and patients continued with a median duration of 28 h. Based on our institutional protocol, a median of two increases in the magnesium sulfate infusion were required to achieve the target serum magnesium concentration.

**Table 2:** Characteristics of magnesium sulfate boluses and infusion in treatment and control groups (*n* = 135).

**Variables**	**Treatment group (*n* = 27)**	**Control group (*n* = 108)**	***p*-value**
	**Number (%) or Median (IQR)**		
**Magnesium sulfate boluses**			
Received magnesium boluses	27 (100.0)	88 (81.5)	**0.01^a^**
Number of magnesium boluses administered	3 (2–4)	1 (1–2)	<**0.01^b^**
Cumulative bolus dose (mg)	4,000.0 (2,750.0–6,000.0)	2,000.0 (1,400.0–2,295.0)	<**0.01^b^**
Cumulative bolus dose (mg/kg)	93.8 (68.8–158.3)	49.6 (34.6–87.1)	<**0.01^b^**
**Magnesium sulfate infusions**			
Rate to achieve the target serum concentration (mg/kg/h)^c^	19.9 (15.8–22.4)	–	–
Duration (hours)	28 (17–58.5)	–	–
Number of infusion rates increased	2 (0–3)	–	–
Number of infusion rates decreased	0 (0–1)	–	–
Cumulative infusion dose (mg)	19,939.0 (9,360.0–56,056.0)	–	–
Cumulative infusion dose (mg/kg)	560.0 (240.0–1,594.0)	–	–

*IQR, Interquartile range*.

a *Fisher's exact test*.

b *Wilcoxon two-sample test*.

c *Target magnesium serum concentration was 4–6 mg/dL*.

Primary and secondary outcomes are shown in Table 3. There was a significant difference in the median PICU LOS between the treatment and control groups, 3.63 vs. 1.09 days, *p* < 0.01. There was no significant difference in the number of patients that received MV between the treatment and control groups, 7 (25.9%) vs. 20 (18.5%), *p* = 0.39. It should be noted that four of these seven patients in the treatment group who received MV, were placed on mechanical ventilation prior to initiation of their magnesium sulfate infusion. Of the patients who received MV, the treatment group had significantly higher median duration of MV compared to control group (4.96 vs. 1.60 days, *p* = 0.02). There was no significant difference in the number of patients in the treatment vs. control group who received non-invasive ventilation, but the median duration of non-invasive ventilation was significantly higher in the treatment vs. control group, *p* < 0.01. There was a significant difference in the number of patients in the treatment vs. control group who received ECMO, 3 (11.1%) vs. 0, *p* < 0.01. For the treatment group, the median duration of ECMO was 2.6 days. Overall, 5 (3.7%) patients expired during their PICU admission, but there was no difference in mortality between groups (*p* = 1.00). One of the patients in treatment group was initiated on ECMO for a total of 12.4 days before expiring.

**Table 3:** Primary and secondary outcomes comparing patients in treatment vs. control groups (*n* = 135).

**Variables**	**Treatment group (*n* = 27)**	**Control group (*n* = 108)**	***p*-value**
	**Number (%) or Median (IQR)**		
**Primary outcomes**			
PICU LOS (days)	3.63 (1.70–7.80)	1.09 (0.66–1.95)	<0.01^a^
MV^b^	7 (25.9)	20 (18.5)	0.39^c^
**Secondary outcomes**			
Duration of MV (days)	4.96 (2–13)	1.60 (0.69–4.75)	0.02^a^
**Non-invasive ventilation**			
Received non-invasive ventilation^d^	27 (100)	98 (90.7)	0.21^e^
Duration of non-invasive ventilation (days)	2.92 (2.04–8.29)	1.44 (0.88–2.67)	<0.01^a^
**ECMO**			
Received ECMO	3 (11.1)	0 (0)	<0.01^e^
Duration on ECMO (days)	2.6 (0.4–12.4)	–	–
Mortality	1 (3.7)	4 (3.7)	1.00^e^

*IQR, Interquartile range; PICU LOS, Pediatric intensive care unit length of stay; MV, Mechanical ventilation; ECMO, Extracorporeal membrane oxygenation*.

a *Wilcoxon two-sample test*.

b *In the treatment group, four out of seven patients were mechanically ventilated before starting magnesium continuous infusion*.

c *Chi-square test*.

d *Non-invasive ventilation included high flow nasal canula, continuous positive airway pressure, and/or bilevel positive airway pressure*.

e *Fisher's Exact test*.

*^f^Exact Chi-square test*.

Table 4 provides a summary of the additional adjunctive status asthmaticus therapies that patients in the treatment and control groups received. There was a significantly higher median duration of continuous albuterol in the treatment vs. control group, 2.08 vs. 0.69 days, *p* < 0.01. The majority (*n* = 133; 98.5%) of patients in the treatment and control groups received IV methylprednisolone with a frequency of every 6 h (*p* = 0.01). Patients in the treatment group had a longer median duration of corticosteroids versus the control group, 5.4 vs. 2.5 days, *p* < 0.01. The patients in the treatment group also had a significantly higher cumulative and daily prednisone equivalent doses than the control group. Other advanced therapies including ketamine, terbutaline and aminophylline were also used significantly more often in patients in the treatment vs. the control group (Table 4). The only significant difference in duration of these advanced therapies between the treatment and control groups was for terbutaline infusions, 2.81 vs. 0.75 days, *p* = 0.01. In addition to this, the actual number of advanced therapies for status asthmaticus were numerically higher in patients in the treatment vs. the control group, though statistical analysis for this could not be performed. Timing of initiation of all therapies is shown in Supplementary Table 1.

**Table 4:** Comparison of medications received by patients in treatment vs. control groups (*n* = 135).

**Variables**	**Treatment group (*n* = 27)**	**Control group (n=108)**	***p*-value**
	**Number (%) or Median (IQR)**		
**Continuous albuterol**			
Duration (days)	2.08 (1.00–5.33)	0.69 (0.42–1.38)	<0.01^a^
**CORTICOSTEROIDS**			
**Agents**			
Methylprednisolone	27 (100)	106 (98.2)	1.00^b^
Prednisolone	11 (40.7)	46 (42.6)	0.86^c^
Prednisone	9 (33.3)	41 (38)	0.66^c^
Dexamethasone	4 (14.8)	26 (24.1)	0.30^c^
Hydrocortisone	1 (3.7)	2 (1.9)	0.49^c^
**Methylprednisolone IV frequency**			
Every 6 h	23 (85.2)	57 (53.8)	0.01^c^
Every 8 h	2 (7.4)	8 (7.6)	
Every 12 h	1 (3.7)	38 (35.9)	
Every 24 h	1 (3.7)	3 (2.8)	
**Dosing regimen**			
Duration (days)	5.4 (3.6–10.3)	2.5 (1.8–3.9)	<0.01^a^
Cumulative dose in mg^d^	693.8 (426.0–1,350.0)	306.3 (178.8–452.5)	<0.01^a^
Cumulative dose in mg/kg^d^	19.0 (13.1–41.3)	7.89 (5.73–11.7)	<0.01^a^
Daily dose in mg/kg/day^d^	3.8 (2.7–4.4)	3.0 (2.3–4.0)	0.02^a^
**ADVANCED THERAPIES OTHER THAN MAGNESIUM SULFATE**			
**Ketamine**			
Received ketamine	11 (40.7)	16 (14.8)	<0.01^c^
Duration (days)	2.5 (0.6–6.0)	1 (0.6–1.3)	0.15^a^
**Terbutaline**			
Received terbutaline	12 (44.4)	8 (7.4)	<0.01^b^
Duration (days)	2.81 (0.83–3.88)	0.75 (0.21–0.88)	0.01^a^
**Aminophylline**			
Received aminophylline	12 (44.4)	3 (2.8)	<0.01^b^
Duration (days)	2.27 (1.29–5.17)	0.96 (0.21–1.38)	0.07
Heliox	3 (11.1)	2 (1.9)	0.05
**Number of advanced therapies administered**			–^e^
Zero	–	86 (79.6)	
One	11 (40.7)	18 (16.7)	
Two	4 (14.8)	3 (2.8)	
Three	5 (18.5)	1 (0.9)	
Four	7 (25.9)	0 (0)	

*IQR, Interquartile range*.

a *Wilcoxon two-sample test*.

b *Fisher's Exact test*.

c *Chi-square test*.

d *Calculated as prednisone equivalent dose*.

e *Statistical analysis was not applicable*.

*^f^Exact Chi-square test*.

Prior to initiation of the magnesium infusion, the median (IQR) baseline concentration was 2.7 (2.3–3.2) mg/dL. The median (IQR) number of serum magnesium concentrations obtained while on an infusion was 5 (3–13). The median (IQR) minimum and maximum magnesium serum concentrations while on the infusion were 3.1 (2.7–3.4) and 4.6 (3.5–5.4) mg/dL. Hypermagnesemia, defined as a serum magnesium concentration >6 mg/dL, was reported in 3 patients (11.1%). None of these patients were noted to have an adverse event. The magnesium infusion was discontinued in one patient, and two patients had their rate of magnesium infusions decreased but continued therapy.

Fourteen (51.9%) patients in the treatment group had an adverse event; four (14.8%) patients experienced ≥1 adverse event. The most identified adverse effect in the treatment group was hypotension, reported in 13 patients (48.1%). Three of these patients with hypotension required an intervention. The magnesium infusions were discontinued in two patients and an IV fluid bolus was given to the third patient. The remaining patients did not require any interventions. Only 1 (3.7%) patient complained of nausea and vomiting and received three doses of ondansetron. There was no documentation of any infusion related reactions or flushing.

### Regression Analysis

A generalized estimating equation logistic and linear regressions were conducted to determine the effect of various factors (i.e., continuous albuterol duration, cumulative prednisone equivalent dosing, ketamine infusion exposure, and magnesium infusion exposure) on MV requirement and PICU LOS, respectively (Table 5). After adjusting for other variables in the model, the odds of being MV were 19.3 times higher for those that received ketamine infusions (95% CI: 5.40–68.88; *p* < 0.01). In addition, the odds of being MV were 10.9% higher for each mg/kg increase in the prednisone equivalent dosing (OR 1.09; 95% CI: 1.03–1.17; *p* < 0.01). Use of mechanical ventilation and duration of continuous albuterol were not independently associated with MV. Use of magnesium and ketamine infusions, and continuous albuterol duration were not associated with an increase in PICU LOS. After adjusting for other variables in the model, each mg/kg increase in total prednisone equivalent dose corresponded to an increase of 0.128 days in the PICU LOS (95% CI 0.096–0.160; *p* < 0.01).

**Table 5:** Logistic and linear regression models looking at mechanical ventilation requirement and PICU LOS.

**Variables**	**Reference value**	**GEE logistic regression for mechanical ventilation (*****n*** **=** 115^*^)		**Variables**	**Reference value**	**GEE linear regression for PICU length of stay (days) (*****n*** **=** **135)**	
		**Odds ratio (95% confidence interval)**	***p*-value**			**Model parameter estimate (95% confidence interval)**	***p*-value**
Intercept		0.013 (0.003–0.063)	<0.01	Intercept		−0.091 (−0.593 to 0.411)	0.72
Received magnesium infusion (*n* = 23)	No magnesium infusions (*n* = 92)	0.031 (0.001–1.201)	0.06	Received magnesium infusions (*n* = 27)	No magnesium infusions (*n* = 108)	−0.148 (−1.09 to 0.793)	0.76
Received ketamine infusions (*n* = 18)	No ketamine infusions (*n* = 97)	19.294 (5.404–68.882)	<0.01	Received ketamine infusions (*n* = 27)	No ketamine infusions (*n* = 108)	0.852 (−0.219 to 1.92)	0.12
Total prednisone equivalent dose (mg/kg)	Mean = 12.24, any 1-unit increase	1.099 (1.029–1.173)	<0.01	Total prednisone equivalent dose (mg/kg)	Mean = 14.11, any 1-unit increase	0.128 (0.096–0.160)	<0.01
Continuous albuterol duration (days)	Mean = 1.43, any 1-unit increase	1.053 (0.696–1.592)	0.81	Continuous albuterol duration (days)	Mean = 1.54, any 1-unit increase	0.297 (−0.014 to 0.609)	0.06

*Patients who received magnesium were optimally matched 1:4 to those who did not receive magnesium using propensity scores generated on sex, obesity, age at admission, PIM III and PRISM III scores. Propensity scores were used in both models to account for matching process*.

* *Four patients received magnesium after mechanical ventilation and were excluded from the logistic regression model along with their matched controls*.

### Subgroup Analysis

A subgroup analysis was performed to compare the outcomes of the patients in the treatment group who received magnesium infusions as their first advanced therapy (*n* = 16) vs. those who received magnesium infusions after another advanced therapy (i.e., 2nd, 3rd, or 4th line) (*n* = 11) (Supplementary Table 2). There were no significant differences in the demographics, primary, and secondary outcomes between groups. There were no significant differences in the number of magnesium boluses, total mg/dose, and mg/kg of magnesium boluses, but there was a significant difference in the magnesium infusion rate and duration between groups. There was a significant difference in the median number of advanced therapies administered between those who received magnesium infusions as their first advanced therapies vs. those who received it in addition to another advanced therapy, 1.0 vs. 3.0, *p* < 0.01.

## Discussion

To our knowledge, this is the first study evaluating both clinical outcomes and adverse events in children receiving magnesium sulfate infusions for ≥4 h for refractory status asthmaticus. Our study noted that the use of magnesium sulfate infusions was not associated with lower odds of MV requirement or PICU LOS when controlling for various independent covariates. In addition, we noted that 14 (51.9%) patients in the treatment group had ≥1 adverse event(s) while on their magnesium sulfate infusion. Several studies have evaluated the clinical outcomes of magnesium sulfate boluses (25–75 mg/kg over 20 min) in the ED setting in children with acute asthma exacerbation, but there are limited reports evaluating impact of magnesium sulfate infusions in children with refractory status asthmaticus (2–5).

Our study focused on relevant outcomes in the PICU including PICU LOS and MV requirement/duration. Of the previously mentioned eight studies in children with refractory status asthmaticus (*n* = 447) evaluating the efficacy and/or safety of magnesium sulfate infusions over >1 h, only three of these studies (*n* = 186; 41.6%) evaluated clinical outcomes (6–13). Only one study by Irazuzta and colleagues evaluated a clinical outcome relevant to our study population (11). They prospectively evaluated the use of a prolonged IV magnesium infusion of 50 mg/kg over 1 h (*n* = 19) vs. an infusion of 50 mg/kg/h over 4 h in the ED setting (11). They found a significantly shorter mean ED LOS with the prolonged infusion vs. the bolus group (34.1 ± 19.5 vs. 48.1 ± 18.7 h, *p* = 0.01). While this study did not assess outcomes in the PICU, the impact on overall LOS is more relevant to our study's findings as we noted a significant increase in PICU LOS in the treatment vs. the control group, 3.63 vs. 1.09 days, *p* < 0.01. However, unlike their study, we controlled for covariates, and in this analysis, magnesium infusions were not associated with increased PICU LOS or MV requirement.

Our study also assessed other secondary outcomes including non-invasive ventilation requirement/duration, ECMO requirement/duration and mortality between groups. We noted no statistical difference in the number of patients in the treatment vs. the control group who received non-invasive ventilation. However, we found a significant difference in the median duration of non-invasive ventilation between the treatment and control groups, 2.92 vs. 1.44 days, *p* < 0.01. Several studies have evaluated the use of non-invasive ventilation in children with status asthmaticus, but it is difficult to compare our study to these reports (21, 22). In our study, we noted a significant difference in the number of patients in the treatment vs. the control group who received ECMO during their PICU stay, 3 (11.1%) vs. 0, *p* < 0.01. For these patients in the treatment group, the median (IQR) duration was 2.6 days (0.4–12.4) or 62.4 h (9.6–297.6). Several studies have reported the use of ECMO for refractory status asthmaticus in children, with a median duration of ECMO of 93–144 h and survival rate of 95–100% (23–25). One of our patients in the treatment group who was initiated on ECMO expired after a prolonged ECMO run of 297.6 h. It is difficult to comment on the impact of magnesium sulfate infusions on the requirement of ECMO given our small sample size and retrospective study design. Overall, we noted that 3.7% of patients in the treatment and control groups expired during their PICU admission, but no difference in mortality was noted between the groups. Other studies evaluating the mortality in children with status asthmaticus have noted a comparable mortality rate to our study of 3.4–4.3% (21, 26).

To account for the impact of other therapies on clinical outcomes, we also collected continuous albuterol duration, prednisone equivalent dosing, and receipt of additional advanced therapies (e.g., aminophylline, terbutaline, ketamine and heliox). We noted a significant difference in the median duration of continuous albuterol between the treatment and the control groups, 2.08 vs. 0.69 days, *p* < 0.01. However, continuous albuterol duration was not significantly associated with the odds of MV requirement or PICU LOS. Several other studies in children with status asthmaticus have assessed the duration of continuous albuterol as one of their primary outcomes; it is difficult to compare our study to these studies given that these studies did not employ prolonged magnesium infusions (21, 27). The treatment group had a significantly higher duration and cumulative and daily prednisone equivalent dosing compared with the control group. It is difficult to compare these findings given that initiation and dosing of corticosteroids was at prescriber discretion. A paucity of data exists on the dosing and impact of corticosteroids on outcomes in children with status asthmaticus. According to one study, many intensivists have been reported to utilize 2–4 times higher corticosteroid dosing compared to the published guidelines for children with asthma exacerbations (28). To account for confounding variables including the cumulative prednisone equivalent dosing, logistic and linear regressions were conducted to assess the odds of MV requirement and factors associated with PICU LOS, respectively. Higher cumulative prednisone equivalent dosing was associated with a higher MV requirement and longer PICU LOS. Previous studies have shown an association between the use of corticosteroids and the development of ICU-acquired weakness; thus, one plausible explanation is that patients receiving a higher cumulative prednisone equivalent dosing had a prolonged duration in MV duration and a corresponding increase in PICU LOS (29).

We also collected additional advanced therapies that the treatment and control groups received. In our study, we found a higher number of patients in the treatment group received an additional advanced therapy. Several previous studies have assessed outcomes in children receiving these advanced therapies (22, 30, 31). However, these authors only assessed outcomes of patients receiving one advanced therapy whereas we evaluated outcomes of children receiving more than one advanced therapy (22, 30, 31). One recent study by Stulce et al. evaluated outcomes in 1,144 children with status asthmaticus, in the Pediatric Health Information System database from 2016 to 2019, that received terbutaline and aminophylline as a second-tier therapy (29). They found a significantly higher odds of intubation and mechanical ventilation in African American children receiving terbutaline vs. those receiving aminophylline (OR, 12.41; 95% CI: 1.61–95.0). This study did not include any information on the number of children receiving prolonged magnesium infusions, so it is difficult to compare to our study. To account for the impact of these advanced therapies on clinical outcomes, we included the number of children receiving ketamine infusions in regression analyses. Both ketamine and magnesium infusions were received by 27 children. Due to the limited numbers of patients receiving heliox, terbutaline, and aminophylline infusions, we were unable to include them in the analyses. After controlling for various covariates, we noted that ketamine infusions were associated with 19.3 times higher odds of MV requirement but had no impact on PICU LOS. It is difficult to completely explain these findings, but it may reflect that ketamine infusions have been employed as a treatment for status asthmaticus and a sedative in patients receiving non-invasive and invasive mechanical ventilation (32).

All the patients who received magnesium sulfate infusions, were initiated at a rate of 15 mg/kg/h and were titrated to a goal serum magnesium concentration of 4–6 mg/dL. We noted a median duration of 28 h, and the median dose to achieve a serum concentration of 4–6 mg/dL was 19.9 mg/kg/h. Five previous studies including 261 children reported on the use of prolonged magnesium infusions over ≥4 h (8–10, 12, 13). It should be noted that all these reports utilized different dosing regimens, but they all utilized the same serum concentration goal of 4–6 mg/dL (33). Since the median duration of magnesium infusions in our study was 28 h, our findings can be compared to two previous reports that described the use of prolonged magnesium infusions over >24 h (12, 13). Patients in our study received comparable dosing and duration to those noted in these studies, with a median infusion rate of 18.4–25 mg/kg/h for a total duration of 53.4–177.5 h. Out of the five studies evaluating magnesium serum concentrations with prolonged magnesium infusions, only three patients (1.1%) developed hypermagnesemia, defined as a concentration >6 mg/dL (8–10, 12, 13). In these reports, no adverse events were attributed to magnesium infusion (13). We noted that 3 (11.1%) patients in the treatment group developed hypermagnesemia. While the percentage of children with hypermagnesemia in our study was higher than these previous reports, we also noted that the supratherapeutic concentrations were not associated with adverse events.

We noted 14 (51.9%) patients in the treatment group had an adverse event, most common adverse events included hypotension (*n* = 13; 41.8%) and nausea/vomiting (*n* = 1; 3.7%). Only two studies evaluating prolonged magnesium infusions reported adverse events (12, 13). The incidence of hypotension in our study was similar to the rate reported by Graff et al. (13). They evaluated adverse events in 154 children receiving magnesium infusions for ≥24 h and noted 170 episodes of hypotension that occurred in 74 of the 154 children (48.1%). Only five (6.8%) of these patients required an intervention with a fluid bolus, reduction in magnesium infusion rate, or initiation of IV maintenance fluids. In our study, we noted that 3 (21.4%) of the 14 patients with hypotension required similar interventions. The only other adverse event we noted in our study was nausea/vomiting in 1 patient. In the two previous studies evaluating magnesium infusions, 7.8% patients (*n* = 35) developed nausea and vomiting; 30 (85.7%) of these patients received ondansetron therapy (12, 13). Similar to these reports, nausea and vomiting in this one patient in our study resolved with ondansetron.

Our study has some limitations. First, the nature of retrospective design may have resulted in some missing or undocumented information. As a result, we were not able to establish a causal relationship between the outcomes and magnesium infusions. To account for this, we did employ the use of regression analyses to evaluate the factors associated with MV requirement and PICU LOS. Second, this study was conducted at a single center. As a result, the results may not be generalizable to other institutions. Third, our study included a limited sample size of children who received prolonged magnesium infusions. However, our sample size of the treatment group was comparable to many other reports analyzing the use of prolonged magnesium infusions in children with status asthmaticus (8–12). Fourth, our institution's refractory status asthmaticus protocol provided guidance on dosing and monitoring of advanced therapies. However, the selection of the therapies was at discretion of the providers. Therefore, we were unable to determine how the selection of advanced therapies may have affected the clinical outcomes. To account for the order of advanced therapies, we conducted a subgroup analysis comparing patients in the treatment group who received magnesium infusions as their first advanced therapy vs. those who received magnesium infusions after another advanced therapy (i.e., 2nd, 3rd, or 4th line). We found no difference in clinical outcomes between groups, but our sample size was small and further exploration is needed.

In conclusion, magnesium sulfate infusions were not associated with MV requirement or PICU LOS when controlling for covariates. Higher prednisone equivalent dosing was associated with an increase in MV requirement and PICU LOS. Overall, 14 (51.9%) patients in the treatment group developed an adverse event but were not associated with hypermagnesemia. Further prospective studies are needed to compare the impact of prolonged magnesium infusions vs. other advanced therapies on outcomes in children with refractory status asthmaticus.

## Data Availability Statement

The raw data supporting the conclusions of this article will be made available by the authors, without undue reservation.

## Ethics Statement

The studies involving human participants were reviewed and approved by the University of Oklahoma Institutional Review Board. Written informed consent from the participants' legal guardian/next of kin was not required to participate in this study in accordance with the national legislation and the institutional requirements.

## Author Contributions

KWT collected the clinical data and drafted this manuscript. SBN performed the statistical analysis for this study. PNJ, JLM, SBN, and NG critically revised the manuscript and approved the final version. All authors contributed to the article and approved the submitted version.

## Conflict of Interest

The authors declare that the research was conducted in the absence of any commercial or financial relationships that could be construed as a potential conflict of interest.

## Publisher's Note

All claims expressed in this article are solely those of the authors and do not necessarily represent those of their affiliated organizations, or those of the publisher, the editors and the reviewers. Any product that may be evaluated in this article, or claim that may be made by its manufacturer, is not guaranteed or endorsed by the publisher.
