# Supplementary material for: Efficacy and Safety of Prolonged Magnesium Sulfate Infusions in Children With Refractory Status Asthmaticus
Source: Front Pediatr. 2022 Jun 9;10:860921. doi: 10.3389/fped.2022.860921 (PMC9218095; doi:10.3389/fped.2022.860921)
Supplement: Supplementary file 1 [file Table_1.DOCX]

**Supplemental Table 1. Timing of therapies received**

| **Variables** | **Treatment Group (n=27)** | **Control Group (n=108)** | **p-value** |
| --- | --- | --- | --- |
|  | **Number (%) or Median (IQR)** | |  |
| Magnesium sulfate infusion  PICU Day Started from Admission  Started on PICU Day 1  Started on PICU Day 2  Started on PICU Day 3++ | 1 (1-2)  15 (55.6)  8 (29.3)  4 (14.8) | --  --  --  -- | --  -- |
| Mechanical ventilation  PICU Day Started from Admission  Started on PICU Day 1  Started on PICU Day 2  Started on PICU Day 3+ | 1 (1-2)  5 (71.4)  1 (14.3)  1 (14.3) | 1 (1-1)  16 (80.0)  2 (10.0)  2 (10.0) | 0.621^a^  0.426^b^ |
| Non-invasive ventilation  PICU Day Started from Admission  Prior to PICU Admission Started on PICU Day 1  Started on PICU Day 2  Started on PICU Day 3+ | 1 (1-3)  0 (0)  9 (70.4)  0 (0)  8 (29.6) | 1 (1-1)  1 (1.0)  73 (74.5)  16 (16.3)  8 (8.2) | 0.287^a^  **0.025^b^** |
| Corticosteroids  PICU Day Started from Admission  Started prior to PICU Admission  Started on PICU Day 1  Started on PICU Day 2  Started on PICU Day 3 | 1 (1-1)  2 (7.4) 22 (81.5) 3 (11.1) 0 (0) | 1 (1-1)  8 (7.4) 85 (78.7)  14 (13.0) 1 (0.9) | 0.765^a^  1.00^c^ |
| Ketamine  PICU Day Started from Admission  Started on PICU Day 1  Started on PICU Day 2  Started on PICU Day 3+ | 1 (1-3)  7 (63.6)  1 (9.1)  3 (27.3) | 1 (1-2)  11 (68.8)  2 (12.5)  3 (18.8) | 0.525^a^  0.826^b^ |
| Terbutaline  PICU Day Started from Admission  Started on PICU Day 1  Started on PICU Day 2  Started on PICU Day 3+ | 1.5 (1-2)  6 (50.0)  4 (33.3)  2 (16.7) | 1 (1-1)  7 (77.8)  2 (22.2)  0 (0) | 0.173^a^  0.708^b^ |
| Aminophylline  PICU Day Started from Admission  Started on PICU Day 1  Started on PICU Day 2  Started on PICU Day 3+ | 1.5 (1.0-4.5)  6 (50.0)  2 (16.7)  4 (33.3) | 2 (1-2)  1 (33.3)  2 (66.7)  0 (0) | 0.939^a^  0.556^b^ |

Abbreviations: IQR = Interquartile range; PICU = Pediatric intensive care unit

^a^Wilcoxon two-sample test; ^b^Chi-square test; ^c^Exact Chi-square test
