# Supplementary material for: Efficacy and Safety of Prolonged Magnesium Sulfate Infusions in Children With Refractory Status Asthmaticus
Source: Front Pediatr. 2022 Jun 9;10:860921. doi: 10.3389/fped.2022.860921 (PMC9218095; doi:10.3389/fped.2022.860921)
Supplement: Supplementary file 2 [file Table_2.DOCX]

**Supplemental Table 2. Comparison of demographics and clinical outcomes in patients who received continuous magnesium infusion as 1^st^ advanced therapy (n=16) versus those who did not (n=11)**

| **Variables** | **Magnesium Sulfate Infusion as 1st Advanced Therapy (n=16)** | **Magnesium Sulfate Infusion Not as 1st Advanced Therapy (n=11)** | **p-value** |
| --- | --- | --- | --- |
|  | **Number (%) or Median (IQR)** | |  |
| Age (years) | 9.14 (7.21 - 11.2) | 7.34 (5.62 - 13.5) | 0.748^a^ |
| Males | 8 (50.0) | 8 (72.7) | 0.427^b^ |
| BMI^c^  BMI (kg/m2)  BMI percentile for age and sex | 19.9 (16.4-23.4)  83.2 (53.8-97.1) | 18.0 (17.1-22.6)  84.8 (65.8-89.9) | 0.863^a^  1.00^a^ |
| Weight-for-length percentile | -- | 99.9^d^ | --^e^ |
| Obesity status | 6 (37.5) | 3 (27.3) | 0.692^b^ |
| Mortality Scores  PIM III scores  PRISM III scores | -4.67 (-6.13 to -4.55)  3 (0-8.5) | -4.65 (-6.12 to -4.52)  5 (2-11) | 0.824^a^  0.377^a^ |
| Race/ethnicity  African American  White/Caucasian  American Indian/Alaska Native  Hispanic  Asian American  Mixed  Unknown/Unspecified | 9 (56.3)  4 (25.0)  2 (12.5)  0 (0)  0 (0)  1 (6.3)  0 (0) | 6 (54.6)  2 (18.2)  0 (0)  2 (18.2)  0 (0)  1 (9.1)  0 (0) | 0.479^f^ |
| ***Primary Outcomes*** |  |  |  |
| Use of mechanical ventilation | 3 (18.8) | 4 (36.4) | 0.391^b^ |
| PICU LOS (days) | 3.3 (1.52-6.58) | 6.88 (2.91-10.5) | 0.120^a^ |
| ***Secondary Outcomes*** | | | |
| Mechanical ventilation  Duration (days)  PICU day initiated | 4.0 (2.0-26.3)  2 (1-13) | 6.2 (3.5-10.3)  1 (1-1) | 0.860^a^  0.123^a^ |
| Non-invasive ventilation  Received non-invasive ventilation^g^  PICU day initiated  Duration of non-invasive ventilation (days) | 16 (100.0)  1 (1-1)  2.9 (2.1-7.0) | 11 (100.0)  3 (1-6)  3.0 (1.7-8.3) | --^e^  **0.026^a^**  0.786^a^ |
| ECMO  Received ECMO  Duration on ECMO (days) | 2 (12.5)  1.5 (0.95-2.1) | 1 (9.1)  12.4 | 1.00^b^  --^e^ |
| Mortality | 1 (6.3) | 0 (0.0) | 1.00^b^ |

**Supplemental Table 2. Continued**

| **Variables** | **Magnesium Sulfate Infusion as 1st Advanced Therapy (n=16)** | **Magnesium Sulfate Infusion Not as 1st Advanced Therapy (n=11)** | **p-value** |
| --- | --- | --- | --- |
|  | **Number (%) or Median (IQR)** | |  |
| ***Magnesium Sulfate Dosage Regimen*** | | | |
| Magnesium sulfate boluses  Received magnesium boluses  Number of magnesium boluses administered  Cumulative bolus dose (mg)  Cumulative bolus dose (mg/kg) | 16 (100.0)  2 (2-3)  3525.0 (5325.0)  86.7 (64.4-123.9) | 11 (100.00)  3 (3-5)  4750.0 (3000.0-8000.0)  125.0 (87.3-281.8) | --^e^  0.132^a^  0.459^a^  0.217^a^ |
| Magnesium sulfate infusions  Rate to achieve the target serum concentration (mg/kg/h)^c^  Duration (hours)  Number of infusion rates increased  Number of infusion rates decreased  Cumulative infusion dose (mg)  Cumulative infusion dose (mg/kg)  PICU day initiated | 18.2 (15.0-20.0)  20.0 (11.5-37.0)  1 (1-2)  1 (1-3)  16062.0 (4811.0-28492.5)  310.0 (152.5-777.5)  1 (1-2) | 20.5 (17.1-29.6)  68.0 (26.0-119.0)  3 (2-3)  1.5 (1-4)  30415.0 (16613.7-63760.0  1594.0 (447.0-2765.0)  2 (1-2) | **0.006^a^**  **0.008^a^**  **0.009^a^**  0.463^a^  **0.036^a^**  **0.006^a^**  0.425^a^ |
| ***Continuous albuterol*** | | | |
| Duration (days) | 1.4 (0.9-3.7) | 3.5 (1.8-6.9) | 0.132^d^ |
| ***Corticosteroids*** | | | |
| Agents  Methylprednisolone  Prednisolone  Prednisone  Dexamethasone  Hydrocortisone | 16 (100.0)  8 (50.0)  4 (25.0)  3 (18.8)  1 (6.3) | 11 (100.0)  3 (27.3)  5 (45.5)  1 (9.1)  0 | --^e^  0.427^b^  0.411^b^  0.624^b^  1.00^b^ |
| ***Corticosteroids***  PICU day initiated | 1 (1-1) | 1 (1-1) | 0.636^a^ |
| Methylprednisolone IV frequency in the PICU  Every 6 hours  Every 8 hours  Every 12 hours  Every 24 hours | 13 (81.3)  1 (6.3)  1 (6.3)  1 (6.3) | 10 (90.9)  1 (9.1)  0  0 | 1.00^b^ |
| Dosing regimen  Duration (days)  Cumulative dose in mg  Cumulative dose in mg/kg | 4.9 (2.8-9.4)  846.9 (420.0-1964.0)  17.9 (11.8-26.8) | 8.7 (4.5-11.0)  579.0 (455.0-11964.0)  19.8 (17.4-49.1) | 0.227^a^  0.941^b^  0.289^b^ |

**Supplemental Table 2. Continued**

| **Variables** | **Magnesium Sulfate Infusion as 1st Advanced Therapy (n=16)** | **Magnesium Sulfate Infusion Not as 1st Advanced Therapy (n=11)** | **p-value** |
| --- | --- | --- | --- |
|  | **Number (%) or Median (IQR)** | |  |
| ***Advanced therapies other than magnesium sulfate*** | | | |
| Ketamine  Received ketamine  PICU day initiated  Duration (days) | 4 (25.0)  1.5 (1-10) 3.3 (2.4-11.8) | 7 (63.6)  1 (1-3)  1.6 (0.3-6.0) | 0.061^b^  0.584^a^  0.395^a^ |
| Terbutaline  Received terbutaline  PICU day initiated  Duration (days) | 4 (25.0)  2 (1.5-10.5)  2.5 (1.3-5.7) | 8 (72.7)  1 (1-2)  2.9 (0.7-3.9) | **0.022^b^**  0.270^a^  0.932^a^ |
| Aminophylline  Received aminophylline  PICU day initiated  Duration (days) | 4 (25.0)  1.5 (1-10.5)  2.0 (0.7-5.3) | 8 (72.7)  1.5 (1-4.5)  2.3 (1.7-5.2) | **0.022^b^**  1.00^a^  0.799^a^ |
| Heliox | 1 (6.3) | 2 (18.2) | 0.549b |
| Number of advanced therapies administered  One  Two  Three  Four | 11 (68.8)  1 (6.3)  1 (6.3)  3 (18.8) | 0  3 (27.3)  4 (36.4)  4 (36.4) | **0.002^f^** |
| Number of advanced therapies | 1.0 (1.0-2.5) | 3.0 (2.0-4.0) | **0.005^a^** |

Abbreviations: IQR = Interquartile range; BMI = Body Mass Index; PIM III = Pediatric Index of Mortality III; PRISM III = Pediatric Risk of Mortality III; PICU = Pediatric Intensive Care Unit; LOS = Length of stay; ECMO = Extracorporeal Membrane Oxygenation

^a^Wilcoxon two-sample test; ^b^Fisher’s Exact test; ^c^BMI assessed in 16 patients in the treatment group and 10 patients in the control group (>2 years of age); ^d^Weight-for-length assessed in one patient in the control group who was <2 years of age; ^e^Statistical analysis not applicable; ^f^Chi-square test; ^g^Non-invasive ventilation included high flow nasal cannula, continuous positive airway pressure, and/or bilevel positive airway pressure; ^h^Exact Chi-square test
