# Supplementary material for: Efficacy and Safety of Prolonged Magnesium Sulfate Infusions in Children With Refractory Status Asthmaticus
Source: Front Pediatr. 2022 Jun 9;10:860921. doi: 10.3389/fped.2022.860921 (PMC9218095; doi:10.3389/fped.2022.860921)
Supplement: Supplementary file 3 [file Table_3.DOCX]

**Supplementary Appendix I**

**Magnesium Protocol**

- Magnesium IV bolus:
  - 25 mg/kg (maximum 2 grams) every 4 hours as needed for magnesium < 3.5 mg/dL
- Magnesium IV infusion:
  - < 40 kg: 15 mg/kg/h
  - ≥ 40 kg: 10 mg/kg/h
- Titrated based on q4h magnesium concentrations as follows:

| **Magnesium Concentrations (mg/dL)** | **Action** |
| --- | --- |
| <3.5 | Bolus 25 mg/kg x 1 and increase infusion by 5 mg/kg/h |
| ≥3.5 to < 4 | Increase infusion by 5 mg/kg/h |
| ≥4 to < 5 | Continue current infusion |
| ≥5 with no signs of magnesium toxicity | Decrease infusion by 5 mg/kg/h |
| ≥6 or ≥ 5 with signs of magnesium toxicity | Stop infusion, notify house officer, re-assess concentration in 2 hours |
